# Healthfulness of Atlanta

**Published:** 1858-07

**Authors:** F. D. Thurman

**Affiliations:** Dentist, Atlanta, Georgia


### ARTICLE II.

*Healthfulness of Atlanta.* By F. D. Thurman, Dentist, Atlanta, Georgia.

Many fortunes have been spent and families reduced to want, in endeavoring to discover perpetual motion, and yet it is not discovered. Its aspirants will occasionally look cheerful and sanguine, with intermediate turns of gloom and despair.

For a time, it seems that the long levers must rule the short ones, and he is obliged to succeed. But, after he spends a few more years in close application and the pleasures of anticipation, he begins sorrowfully to see that nature's laws are so well balanced, that every fraction of power gained in one part of the machine is exactly balanced by a loss of time in another part, and *vice versa.* He is now ready to agree with the Poet, that—

"The world is harmoniously confused."

And thus it is, all things in nature are subject to advantages and disadvantages. The North has the advantage of the South, in not being perplexed with slaves. The South has the advantage of the North in having negroes to work for them. One man has the advantage of education, another of honesty, another enjoys both, another is deprived of both. One city has the advantage of navigable streams, another of railroads, another of both, and another is deprived of both. One enjoys wealth, another is blest with health.

Now the city that enjoys the greatest number and weight of advantages is most to be desired. As to the railroads of Atlanta, comment is unnecessary, they speak for themselves. But I think that its healthfulness is too often overlooked.

Health is the greatest blessing that mortal man can enjoy; deprive him of health, and you deprive him of all the sweets of life. Though other blessings may be heaped in profusion upon him, they afford no consolation. These are facts that all are ready to admit, yet few practice upon them. We seldom think of taking care of ourselves, until there is nothing left of us worth taking care of. But to those who appreciate the value of a healthy situation, I would say come to Atlanta; where all classes prosper except Physicians, and even they enjoy the privilege of exulting over their rosy cheeks.

Atlanta is situated on seven hills, with seven or more springs rising within the corporation, and flowing off in *every direction,* some being gathered around to one side, wend their way to the Atlantic; whilst others flowing North, South and West, seek their level in the Gulf of Mexico. Though the city is well watered with springs, not a solitary stream, not even of the smallest size, passes through or near any part of it that does not rise inside of the corporation line. All of this, of course, shows an elevated situation.

The hills are so beautifully rounded off that instead of marring, they add greatly to the beauty of the place. They are so well shaped that notwithstanding they are flat enough for beauty, they are round enough not to leave a solitary spot on the hills or in the valleys for the retention of stagnant water; but the city is literally washed by every rain.

Our well water is all good; nearly every man will tell you that his water is really the best in town.

Our atmosphere is pure and healthful, and as Professor Means says, "We are too high up for malarious diseases, such as fevers of various kinds, and too low down for mountainous diseases, such as consumptions, rheumatisms," &c. In fact, I might say that we labor under but one disadvantage, and that is not a permanent one. It is a well known fact that in all of our populous cities, the poor classes (owing to causes not necessary to be mentioned here) are always more subject to diseases, than the more wealthy. To this defect we plead guilty. I suppose that no city of the same size has a greater proportion of those who are not supplied with worldly goods. But notwithstanding this disadvantage, surprising to say, our mortality is computed at only one and a half per centum per annum, including rail road accidents.

And as I remarked above, this defect is not a permanent one; we are growing rich; and the rich are moving in for their health; and oh! how I love to hear the latter speak of their dear little children, which a short time since were brought here with pale, sickly faces, and now blooming with health.

All these are stubborn facts, and all speak well of our thriving city, which has grown contrary to the expectations of every one, and in my humble opinion is destined ere long to increase their surprise to astonishment. More anon.

